# Development and external validation of a musculoskeletal ultrasound-based prediction model for limited shoulder range of motion in nursing staff with neck–shoulder pain: a cross-sectional study

**DOI:** 10.3389/fpubh.2026.1830887

**Published:** 2026-05-04

**Authors:** Rui Zhao, Hongyan Zhang, Jingfeng Zhang, Lei Zhang, Yanping Wan

**Affiliations:** 1 Department of Anorectal, Baoji Central Hospital, Baoji, China; 2 Department of Respiratory and Critical Care Medicine, Baoji Hospital of Traditional Chinese Medicine, Baoji, China; 3 Department of Ultrasound Medicine, Baoji High-Tech Hospital, Baoji, China; 4 Department of Radiology, Baoji High-Tech Hospital, Baoji, China; 5 Department of Radiology, The First Affiliated Hospital of Xi’an Jiaotong University, Xi’an, China

**Keywords:** external validation, limited shoulder range of motion, musculoskeletal ultrasound, neck–shoulder pain, nomogram, nurses, prediction model, risk factors

## Abstract

**Background:**

Nurses with neck–shoulder pain are at high risk of developing limited shoulder range of motion (ROM), but early prediction tools are lacking. This study aimed to identify risk factors using clinical data and musculoskeletal ultrasound (MSKUS), and to develop and validate a prediction model.

**Methods:**

A total of 210 nurses with neck–shoulder pain (100 with limited ROM, 110 with normal ROM) from a tertiary hospital were enrolled. Univariate and multivariate logistic regression analyses were performed to establish a prediction model, which was externally validated in 63 additional nurses.

**Results:**

Multivariate analysis identified age, average sleep duration on workdays (ASDW), daily overhead arm duration (DOAD), glenohumeral joint effusion (GLE), subacromial-subdeltoid bursa effusion (SASDBE), and long head of biceps tendon sheath effusion (LHBTE) as independent risk factors. The prediction model demonstrated excellent discrimination with an area under the ROC curve (AUC) of 0.956, sensitivity of 0.910, and specificity of 0.864. External validation yielded an AUC of 0.938, sensitivity of 0.833, and specificity of 0.821.

**Conclusion:**

The model incorporating age, ASDW, DOAD, GLE, SASDBE, and LHBTE shows excellent predictive performance for limited shoulder ROM in nursing staff with neck–shoulder pain.

## 1 Introduction

Nurses, the largest healthcare occupational group, are at higher risk for musculoskeletal disorders than other medical professionals (1). Among these conditions, neck–shoulder pain demonstrates a notably high prevalence, accounting for approximately 16–26% of all musculoskeletal diseases (2, 3). In China, factors including the substantial population base, aging demographics, healthcare workforce shortages, and patient preference for tertiary hospitals contribute to an elevated incidence of neck–shoulder pain among nurses with ≥3 years of work experience in such institutions (4, 5).

Neck–shoulder pain arises from various pathophysiological mechanisms, including cervical and shoulder muscle contraction, ischemia, and spasm, typically manifesting as persistent regional stiffness and discomfort; severe cases may progress to limited shoulder range of motion (ROM) (6, 7). Risk factors include female sex, BMI, occupational demands, short sleep duration (8, 9), hyperglycemia, thyroid dysfunction, and anxiety (10, 11). Excessive mechanical loading of cervical and shoulder musculature, particularly prolonged daily overhead arm duration (DOAD) (12), may precipitate neck–shoulder pain complicated by functional impairment. Early risk assessment is critical given this high prevalence and associated disability. Previous studies relied on subjective questionnaires, lacking objective confirmation. Notably, musculoskeletal ultrasound (MSKUS) enables accurate identification of pathologies underlying limited shoulder ROM, including rotator cuff tears, tendinopathy, bursal effusion, and long head of biceps tendon sheath effusion (LHBTE) (13).

This study analyzed clinical characteristics and MSKUS findings from nursing staff with neck–shoulder pain to identify risk factors for limited shoulder ROM, subsequently developing and externally validating a prediction model. The objective was to develop an evidence-based tool for personalized risk stratification to prevent functional decline in this essential workforce.

## 2 Methods

### 2.1 Study design

This cross-sectional study enrolled female nurses with neck–shoulder pain identified during routine health examinations at a tertiary hospital in Baoji City, China, between January 2023 and January 2025. A total of 210 nurses were recruited using convenience sampling for model development and subsequently classified into two groups based on shoulder mobility: limited shoulder range of motion (ROM) group and normal shoulder ROM group. To minimize selection bias associated with convenience sampling, the following measures were implemented: (1) recruitment was conducted across all medical and surgical departments to ensure diverse clinical settings; (2) consecutive eligible nurses were approached during routine health examinations to reduce self-selection bias; (3) standardized inclusion and exclusion criteria were strictly applied by two independent investigators; and (4) the validation cohort was prospectively recruited from the same hospital but during a different time period (February–July 2025) to assess temporal stability.

Inclusion criteria were as follows: (i) unilateral neck–shoulder pain; (ii) nursing professionals with ≥3 years of experience in medical or surgical departments; and (iii) no history of shoulder or neck–shoulder pain treatment within the preceding month.

Exclusion criteria comprised: (i) organic cervical or shoulder pathologies, including trauma, surgery, rotator cuff injury, nerve entrapment syndromes, shoulder arthritis, cervical disc disorders, or cervical spondylosis; and (ii) systemic diseases affecting the brain, lungs, heart, liver, or kidneys that might cause referred neck–shoulder pain.

Additionally, 63 nursing personnel meeting identical inclusion and exclusion criteria were prospectively enrolled between February 2025 and July 2025 for external model validation. Sample size was estimated using the events-per-variable principle (14), each candidate predictor requires at least ten events, where ‘events’ refer to the number of participants with limited shoulder ROM (the smaller outcome group). Based on 12 candidate predictors identified from relevant literature (8–12), and accounting for a 10% attrition rate, the minimum required sample size for the modeling cohort was calculated as 132 participants.

This study protocol was approved by the Ethics Committee of Baoji High-Tech Hospital (Approval No. 2024-021), and written informed consent was obtained from all participants.

### 2.2 Data collection

A comprehensive dataset comprising 12 variables was collected through physical examination and musculoskeletal ultrasound (MSKUS) assessment. General clinical characteristics included: age; body mass index (BMI); daily overhead arm duration (DOAD, defined as ≥10 min daily with the upper arm elevated above the upper ear border); average sleep duration on workdays (ASDW, defined as ≥7 h); department (medical or surgical); affected side (right or left); history of hyperglycemia; anxiety status; and thyroid dysfunction.

Shoulder ROM assessment was performed using a standard goniometer to measure shoulder flexion, abduction, and internal/external rotation angles, following established protocols (15, 16). Limited shoulder ROM was defined as flexion and abduction <180° and internal/external rotation <90°.

MSKUS examination was conducted by a physician with >3 years of MSKUS experience using a GE LOGIQ S8 ultrasound system (GE Healthcare, Milwaukee, WI, USA), adhering to European Society of Musculoskeletal Radiology guidelines (17). Long head of biceps tendon sheath effusion (LHBTE) was assessed by longitudinal placement of the transducer over the intertubercular groove to detect anechoic fluid surrounding the tendon. Subacromial-subdeltoid bursa effusion (SASDBE) was evaluated by positioning the transducer over the greater tuberosity to identify hypoechoic or anechoic fluid accumulation within the bursa. Glenohumeral joint effusion (GLE) was examined with the patient’s hand resting on the contralateral shoulder while placing the transducer posteriorly over the glenohumeral joint space to visualize the posterior recess.

Diagnostic criteria for effusion were as follows: GLE was defined as an anechoic area between the posterior glenoid labrum and the infraspinatus tendon (18); SASDBE was identified by an anechoic band ≥2 mm in depth within the bursa (19); and LHBTE was characterized by an anechoic zone ≥2 mm in width surrounding the tendon within the sheath, with absent color Doppler signal (19).

## 3 Results

### 3.1 Baseline characteristics of the modeling cohort

Among 210 nurses with neck–shoulder pain in the modeling cohort, 100 exhibited limited shoulder ROM and 110 demonstrated normal shoulder ROM. No significant between-group differences were observed in BMI, department, affected side, history of hyperglycemia, anxiety, or thyroid dysfunction (all *p* > 0.05). However, the limited ROM group was significantly older, reported shorter sleep duration, and demonstrated prolonged DOAD compared with the normal ROM group (all *p* < 0.05). MSKUS-assessed GLE, SASDBE, and LHBTE were significantly more prevalent in the limited ROM group (all *p* < 0.05). Detailed statistics are presented in Table 1.

**Table 1:** Comparison of baseline characteristics between groups in the modeling cohort.

Variables	Total (*n* = 210)	Normal ROM (*n* = 110)	Limited ROM (*n* = 100)	Statistic	*p*
Age, *M* (*Q*₁, *Q*₃)	51.000 (41.000, 54.000)	42.000 (40.000, 52.750)	52.000 (51.000, 54.000)	*Z* = −3.305	<0.001
BMI, *M* (*Q*₁, *Q*₃)	24.570 (23.935, 25.725)	24.830 (23.650, 26.310)	24.510 (24.188, 24.795)	*Z* = −1.434	0.152
ASDW, *n* (%)				*χ*^2^ = 30.414	<0.001
≥7 h	129 (61.429)	87 (79.091)	42 (42.000)		
<7 h	81 (38.571)	23 (20.909)	58 (58.000)		
DOAD, *n* (%)				*χ*^2^ = 59.549	<0.001
<10 min	109 (51.905)	85 (77.273)	24 (24.000)		
≥10 min	101 (48.095)	25 (22.727)	76 (76.000)		
GLE, *n* (%)				*χ*^2^ = 76.178	<0.001
Absent	102 (48.571)	85 (77.273)	17 (17.000)		
Present	108 (51.429)	25 (22.727)	83 (83.000)		
SASDBE, *n* (%)				*χ*^2^ = 68.914	<0.001
Absent	92 (43.810)	78 (70.909)	14 (14.000)		
Present	118 (56.190)	32 (29.091)	86 (86.000)		
LHBTE, *n* (%)				*χ*^2^ = 71.429	<0.001
Absent	102 (48.571)	84 (76.364)	18 (18.000)		
Present	108 (51.429)	26 (23.636)	82 (82.000)		
Department, *n* (%)				*χ*^2^ = 1.452	0.228
Medical	134 (63.810)	66 (60.000)	68 (68.000)		
Surgical	76 (36.190)	44 (40.000)	32 (32.000)		
Affected side, *n* (%)				*χ*^2^ = 0.336	0.562
Left	109 (51.905)	55 (50.000)	54 (54.000)		
Right	101 (48.095)	55 (50.000)	46 (46.000)		
Hyperglycemia, *n* (%)				*χ*^2^ = 0.336	0.562
Absent	184 (87.619)	95 (86.364)	89 (89.000)		
Present	26 (12.381)	15 (13.636)	11 (11.000)		
Anxiety, *n* (%)				*χ*^2^ = 0.821	0.365
Absent	187 (89.048)	100 (90.909)	87 (87.000)		
Present	23 (10.952)	10 (9.091)	13 (13.000)		
Thyroid dysfunction, *n* (%)				*χ*^2^ = 0.563	0.453
Absent	148 (70.476)	80 (72.727)	68 (68.000)		
Present	62 (29.524)	30 (27.273)	32 (32.000)		

*Z*, Mann–Whitney test, *χ*^2^, chi-square test; *M*, Median; *Q*₁, 1st Quartile; *Q*₃, 3rd Quartile; SD, standard deviation; BMI, body mass index; DOAD, daily overhead arm duration; ASDW, average sleep duration on workdays; GLE, glenohumeral joint effusion; SASDBE, subacromial subdeltoid bursa effusion; LHBTE, long head of biceps tendon sheath effusion.

### 3.2 Multivariate logistic regression analysis

Variables with statistical significance in univariate analysis were entered as independent variables into a multivariate logistic regression model, with limited shoulder ROM as the dependent variable. Variable coding is detailed in Table 2. The results identified age, ASDW, DOAD, GLE, SASDBE, and LHBTE as independent predictors of limited shoulder ROM (all *p* < 0.05) (Table 3).

**Table 2:** Coding of independent variables.

Variable	Coding
Age	Continuous (years)
ASDW	0 = ≥7 h; 1 = <7 h
DOAD	0 = <10 min; 1 = ≥10 min
GLE	0 = Absent; 1 = Present
SASDBE	0 = Absent; 1 = Present
LHBTE	0 = Absent; 1 = Present

ASDW, average sleep duration on workdays; DOAD, daily overhead arm duration; GLE, glenohumeral joint effusion; SASDBE, subacromial-subdeltoid bursa effusion; LHBTE, long head of biceps tendon sheath effusion.

**Table 3:** Multivariate logistic regression analysis of limited shoulder ROM in nursing staff with neck–shoulder pain.

Variables	*β*	S.E.	Wald *χ*^2^	*p*	OR (95%CI)
Age	0.069	0.029	5.717	0.017	1.071 (1.012 ~ 1.133)
ASDW	1.218	0.530	5.290	0.021	3.381 (1.197 ~ 9.546)
DOAD	1.312	0.501	6.843	0.009	3.712 (1.389 ~ 9.916)
GLE	2.361	0.519	20.657	<0.001	10.603 (3.830 ~ 29.350)
SASDBE	2.149	0.515	17.405	<0.001	8.579 (3.126 ~ 23.548)
LHBTE	2.122	0.498	18.174	<0.001	8.347 (3.146 ~ 22.144)
Constant	−8.021	1.892	17.966	<0.001	0.000

ASDW, average sleep duration on workdays; DOAD, daily overhead arm duration; GLE, glenohumeral joint effusion; SASDBE, subacromial subdeltoid bursa effusion; LHBTE, long head of biceps tendon sheath effusion; OR, odds ratio; CI, confidence interval.

Multicollinearity diagnostics revealed variance inflation factors of 1.064, 1.172, 1.367, 1.361, 1.325, and 1.329, with tolerances of 0.940, 0.854, 0.731, 0.735, 0.755, and 0.752, respectively, indicating no multicollinearity among predictors.

### 3.3 Development and visualization of the prediction model

**Figure 1:**
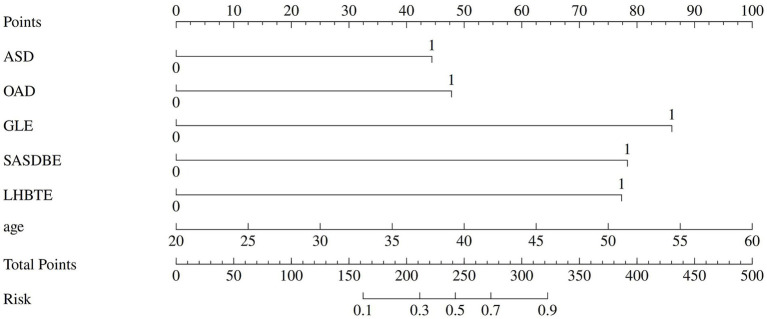
Nomogram for predicting limited shoulder range of motion in nursing staff with neck–shoulder pain. ASDW, average sleep duration on workdays; DOAD, daily overhead arm duration; GLE, glenohumeral joint effusion; SASDBE, subacromial-subdeltoid bursa effusion; LHBTE, long head of biceps tendon sheath effusion.

Based on multivariate logistic regression, the probability of limited shoulder ROM (*p*) was calculated as: Logit (*p*) = −8.021 + 0.069 × Age + 1.218 × ASDW + 1.312 × DOAD + 2.361 × GLE + 2.149 × SASDBE + 2.122 × LHBTE. The Hosmer–Lemeshow test demonstrated adequate model fit (*χ*^2^ = 4.698, *p* = 0.789). The predictive model was visualized as a nomogram (Figure 1).

### 3.4 Model performance evaluation

Receiver operating characteristic (ROC) curve analysis yielded an area under the curve (AUC) of 0.956 (95% CI: 0.930–0.981). At the optimal cutoff value of 0.414 determined by the Youden index (maximizing sensitivity + specificity − 1), the model achieves clinically meaningful discrimination. This threshold corresponds to a predicted probability of approximately 41.4%, above which nurses should be considered high-risk for limited ROM and prioritized for early intervention (e.g., ergonomic adjustment, targeted physiotherapy, or close monitoring). At this cutoff, the model demonstrated sensitivity of 0.910 (95% CI: 0.854–0.966), specificity of 0.864 (95% CI: 0.800–0.928), accuracy of 0.886 (95% CI: 0.835–0.925), positive predictive value of 0.858 (95% CI: 0.792–0.925), and negative predictive value of 0.913 (95% CI: 0.859–0.967). The high negative predictive value (0.913) at this cutoff supports its utility for ruling out significant risk, potentially reducing unnecessary referrals in resource-limited settings.

**Figure 2:**
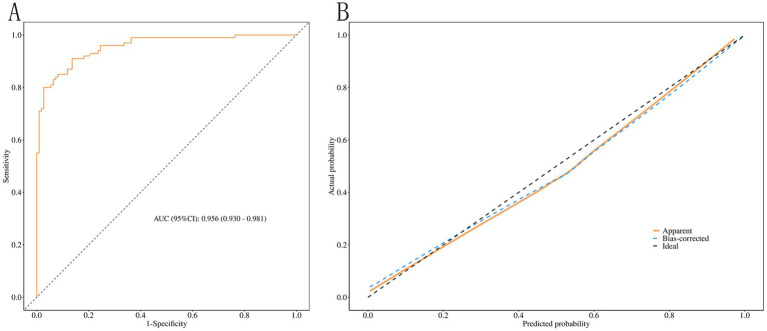
Model performance evaluation in the modeling cohort. **(A)** Receiver operating characteristic (ROC) curve showing an area under the curve (AUC) of 0.956 (95% CI: 0.930–0.981) for predicting limited shoulder ROM. **(B)** Calibration curve comparing predicted probabilities (*x*-axis) with actual observed probabilities (*y*-axis). The dashed diagonal line represents perfect calibration, and the solid line represents the model’s calibration performance.

The calibration curve closely approximated the ideal diagonal, further confirming excellent model fit (Figures 2A,B).

### 3.5 External validation

Clinical and MSKUS data from 63 additional nurses with neck–shoulder pain were prospectively collected between February 2025 and July 2025 for external validation. Baseline characteristics of the modeling and validation cohorts are compared in Table 4. No significant differences were observed between cohorts in any variable (all *p* > 0.05), confirming comparability.

**Table 4:** Comparison of baseline characteristics between modeling and validation cohorts.

Variables	Total (*n* = 273)	0 (*n* = 63)	1 (*n* = 210)	Statistic	*p*
Age, *M* (*Q*₁, *Q*₃)	50.000 (41.000, 54.000)	44.000 (41.000, 53.000)	51.000 (41.000, 54.000)	*Z* = −0.327	0.744
BMI, *M* (*Q*₁, *Q*₃)	24.530 (23.850, 25.490)	24.330 (23.690, 25.370)	24.570 (23.935, 25.725)	*Z* = −1.390	0.165
Limited, *n* (%)				*χ*^2^ = 1.773	0.183
Absent	149 (54.579)	39 (61.905)	110 (52.381)		
Present	124 (45.421)	24 (38.095)	100 (47.619)		
ASDW, *n* (%)				*χ*^2^ = 0.025	0.874
≥7 h	167 (61.172)	38 (60.317)	129 (61.429)		
<7 h	106 (38.828)	25 (39.683)	81 (38.571)		
DOAD, *n* (%)				*χ*^2^ = 0.534	0.465
<10 min	145 (53.114)	36 (57.143)	109 (51.905)		
≥10 min	128 (46.886)	27 (42.857)	101 (48.095)		
GLE, *n* (%)				*χ*^2^ = 0.281	0.596
Absent	135 (49.451)	33 (52.381)	102 (48.571)		
Present	138 (50.549)	30 (47.619)	108 (51.429)		
SASDBE, *n* (%)				*χ*^2^ = 0.570	0.450
Absent	123 (45.055)	31 (49.206)	92 (43.810)		
Present	150 (54.945)	32 (50.794)	118 (56.190)		
LHBTE, *n* (%)				*χ*^2^ = 0.565	0.452
Absent	136 (49.817)	34 (53.968)	102 (48.571)		
Present	137 (50.183)	29 (46.032)	108 (51.429)		
Department, *n* (%)				*χ*^2^ = 0.534	0.465
Medical	171 (62.637)	37 (58.730)	134 (63.810)		
Surgical	102 (37.363)	26 (41.270)	76 (36.190)		
Affected side, *n* (%)				*χ*^2^ = 0.259	0.611
Left	144 (52.747)	35 (55.556)	109 (51.905)		
Right	129 (47.253)	28 (44.444)	101 (48.095)		
Hyperglycemia, *n* (%)				*χ*^2^ = 0.157	0.692
Absent	238 (87.179)	54 (85.714)	184 (87.619)		
Present	35 (12.821)	9 (14.286)	26 (12.381)		
Anxiety, *n* (%)				*χ*^2^ = 0.147	0.702
Absent	242 (88.645)	55 (87.302)	187 (89.048)		
Present	31 (11.355)	8 (12.698)	23 (10.952)		
Thyroid dysfunction, (%)				*χ*^2^ = 0.781	0.377
Absent	196 (71.795)	48 (76.190)	148 (70.476)		
Present	77 (28.205)	15 (23.810)	62 (29.524)		

*Z*, Mann–Whitney test; *χ*^2^, chi-square test; *M*, Median; *Q*₁, 1st Quartile; *Q*₃, 3rd Quartile; ASD, adequate sleep duration; DOAD, daily overhead arm duration; ASDW, average sleep duration on workdays; GLE, glenohumeral joint effusion; SASDBE, subacromial subdeltoid bursa effusion; LHBTE, long head of biceps tendon sheath effusion.

**Figure 3:**
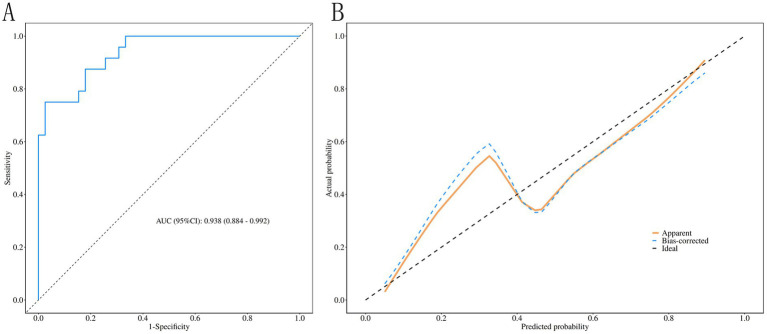
External validation of the prediction model. **(A)** ROC curve for the validation cohort demonstrating an AUC of 0.938 (95% CI: 0.884–0.992). **(B)** Calibration curve for the validation cohort showing close agreement between predicted and observed probabilities, confirming the model’s generalizability.

The validation cohort demonstrated an AUC of 0.938 (95% CI: 0.884–0.992) at the identical cutoff of 0.414, with sensitivity of 0.833 (95% CI: 0.684–0.982), specificity of 0.821 (95% CI: 0.700–0.941), accuracy of 0.825 (95% CI: 0.709–0.909), positive predictive value of 0.741 (95% CI: 0.575–0.906), and negative predictive value of 0.889 (95% CI: 0.786–0.992). The Hosmer–Lemeshow test confirmed adequate fit in the validation cohort (*χ*^2^ = 10.977, *p* = 0.203), with calibration curves closely aligned with ideal predictions (Figures 3A,B).

### 3.6 Clinical utility assessment

**Figure 4:**
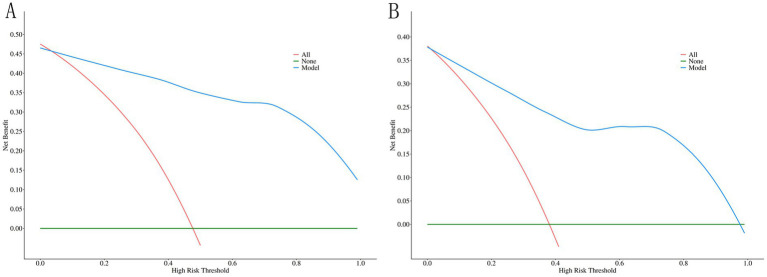
Decision curve analysis evaluating the clinical utility of the prediction model. **(A)** Net benefit curves for the modeling cohort. **(B)** Net benefit curves for the validation cohort.

Decision curve analysis was performed to evaluate clinical net benefit. Net benefit curves for both modeling and validation cohorts consistently exceeded the two extreme curves, indicating significant clinical utility across a broad range of decision thresholds (Figures 4A,B).

## 4 Discussion

### 4.1 Principal findings

This study developed and validated a novel prediction model for limited shoulder ROM in nursing staff with neck–shoulder pain. The final model incorporating six independent predictors—age, ASDW, DOAD, GLE, SASDBE, and LHBTE—demonstrated excellent discrimination (AUC = 0.956) and calibration (Hosmer-Lemeshow test, *p* = 0.789) in the modeling cohort. External validation confirmed robust generalizability with an AUC of 0.938. This is the first study integrating clinical parameters with MSKUS findings to predict functional shoulder impairment in nurses.

The high AUC values (0.956 in the modeling cohort and 0.938 in the validation cohort) might raise concerns regarding potential overfitting. However, several lines of evidence support the robustness and genuine predictive performance of our model: (1) temporal validation: the external validation cohort was recruited prospectively during a different time period (February–July 2025), demonstrating stability across time and ruling out overfitting to specific temporal artifacts; (2) performance consistency: the minimal decrease in AUC between modeling and validation cohorts (ΔAUC = 0.018) indicates genuine predictive signal rather than overfitting, as overfit models typically exhibit substantial performance decay in external validation; (3) decision curve analysis: positive net benefit across clinically relevant threshold probabilities in both cohorts confirms clinical utility beyond mere statistical discrimination; (4) multicollinearity absence: variance inflation factors (VIF) ranging from 1.064 to 1.367 indicate stable, independent coefficient estimates without redundant predictors; and (5) biological plausibility: the identified predictors align with established biomechanical and pathophysiological mechanisms of shoulder dysfunction. These findings collectively suggest that the high AUC reflects true predictive discrimination rather than overfitting.

### 4.2 Comparison with previous literature

The identified risk factors align with established understanding of musculoskeletal disorders in healthcare workers. Advanced age is associated with degenerative shoulder pathologies (20, 21). Our finding that ASDW <7 h increases risk (OR = 3.381) corroborates research linking sleep deprivation with heightened pain sensitivity and impaired musculoskeletal recovery (22, 23). The strong association between DOAD ≥10 min and limited ROM (OR = 3.712) supports ergonomic studies demonstrating that sustained overhead arm positioning exceeds the physiological tolerance of periarticular structures (24, 25).

The MSKUS findings provide novel insights. GLE exhibited the strongest predictive value (OR = 10.603), reflecting capsular inflammation and synovitis (26). Similarly, SASDBE (OR = 8.579) and LHBTE (OR = 8.347) represent subacromial impingement and bicipital tenosynovitis, established precursors to adhesive capsulitis and chronic shoulder dysfunction (27, 28). Clinical examination alone often misses these effusive pathologies (29).

Notably, our model excluded BMI, hyperglycemia, and anxiety—factors previously linked to neck–shoulder pain in the literature (10, 11). This divergence likely reflects a fundamental distinction between pain generation and mechanical function impairment. BMI, hyperglycemia, and anxiety may influence nociceptive processing and pain reporting without necessarily compromising glenohumeral kinematics. In contrast, GLE, SASDBE, and LHBTE represent structural pathologies that physically restrict joint mobility through capsular inflammation, subacromial space narrowing, and tendon sheath effusion—mechanisms that directly constrain range of motion regardless of pain perception. This suggests that while metabolic and psychological factors may initiate or amplify pain signals, structural changes detected by MSKUS constitute the proximate cause of functional limitation in this occupational population.

Several factors specific to nursing staff may explain the predominance of structural over systemic/psychological predictors. First, the homogeneous occupational biomechanical exposure (repetitive overhead patient handling) in this cohort may have overwhelmed individual metabolic variations, rendering BMI and hyperglycemia less discriminating for ROM outcomes. Second, the study population comprised experienced nurses (≥3 years) with established structural adaptations or pathologies, whereas metabolic and psychological factors may exert greater influence during earlier disease stages. Third, MSKUS detects subclinical effusions and structural changes that precede symptomatic pain, capturing the ‘silent’ mechanical deterioration that ultimately determines functional capacity. This temporal sequence—structural damage preceding functional limitation—may explain why imaging biomarkers outweighed systemic factors in predicting ROM rather than pain presence.

### 4.3 Clinical implications

The nomogram offers a practical, non-invasive tool for early identification of nurses at high risk for progressive shoulder disability. Routine MSKUS screening could enable targeted interventions before irreversible functional limitation develops. The model’s high negative predictive value (0.913) suggests utility for ruling out significant risk, potentially reducing unnecessary referrals.

For clinical implementation, healthcare providers should use the nomogram as follows: (1) locate the patient’s age on the age axis and draw vertically upward to determine points; (2) repeat for each predictor (ASDW, DOAD, GLE, SASDBE, LHBTE); (3) sum all points to obtain total points; (4) draw vertically downward from total points to the probability axis to obtain predicted risk. For example, a 50-year-old nurse with ASDW <7 h, DOAD ≥10 min, and positive GLE, SASDBE, LHBTE would accumulate approximately 180 points, corresponding to ~85% predicted probability of limited ROM, warranting immediate intervention. In occupational health practice, we recommend: (i) routine screening for nurses with ≥3 years experience using this nomogram; (ii) immediate ergonomic assessment for those with >40% predicted probability; (iii) quarterly MSKUS monitoring for high-risk individuals; and (iv) workload adjustment for those with progressive functional decline.

Decision curve analysis demonstrated positive net benefit, indicating that this model outperforms both universal intervention and watchful waiting, particularly relevant for resource-limited settings.

### 4.4 Strengths and limitations

Strengths include prospective external validation, standardized MSKUS protocols (17), and objective functional outcomes. The high AUC values in both cohorts suggest genuine predictive utility rather than overfitting.

Several limitations warrant consideration. First, the cross-sectional design precludes definitive causal inference and establishment of temporal sequences. The identified associations between age, ASDE, DOAD, and MSKUS findings with limited ROM represent predictive relationships useful for risk stratification, not etiological causation. We cannot determine whether MSKUS effusions preceded functional decline or resulted from concurrent pathological processes, nor can we establish the temporal order of exposure-outcome relationships. While we recognize this as a fundamental limitation, the value of this prediction model lies in its ability to identify high-risk individuals prospectively, regardless of causal directionality. This distinction is critical: our objective was prognostic prediction rather than etiological inference. Longitudinal follow-up would be required to clarify whether these factors are antecedent causes or concomitant markers of functional impairment. Second, convenience sampling from a single center may limit generalizability. Third, we excluded male nurses due to their small numbers in our institution (comprising <5% of the nursing workforce), precluding assessment of sex-specific biomechanical adaptations and limiting generalizability to male nursing populations. However, several factors support our findings: (a) the study hospital is a tertiary referral center comparable to national statistics (4, 5); (b) recruitment across diverse medical and surgical departments enhanced sample heterogeneity; (c) consecutive enrollment reduced self-selection bias; and (d) the external validation cohort demonstrated consistent performance metrics (AUC = 0.938). Notably, female nurses comprise approximately 92% of the nursing workforce in China (4), suggesting our findings remain applicable to the vast majority of this occupational group, though validation in male nurses and other ethnic/geographic populations is warranted.

Fourth, the validation cohort was relatively small (*n* = 63). However, this sample size is adequate for preliminary external validation according to Riley et al.’s (30) criteria, which recommend a minimum of 100 events in the development cohort and external validation in at least 100 independent participants for stable C-statistic estimation. With 100 events in our modeling cohort and 6 predictors in the final model (EPV = 16.7:1), and given that our validation cohort (*n* = 63) provides sufficient statistical power to detect large discrimination differences (AUC > 0.85) with 80% power at *α* = 0.05, the sample sizes satisfy minimum requirements for prediction model development and preliminary validation. Importantly, baseline characteristics between modeling and validation cohorts were comparable (all *p* > 0.05): age (median 51.0 vs. 44.0 years, *p* = 0.744), BMI (median 24.57 vs. 24.33 kg/m^2^, *p* = 0.165), DOAD distribution (48.1% vs. 42.9% with ≥10 min, *p* = 0.465), ASDW distribution (38.6% vs. 39.7% with <7 h, *p* = 0.874), and MSKUS findings (GLE: 51.4% vs. 47.6%, *p* = 0.596; SASDBE: 56.2% vs. 50.8%, *p* = 0.450; LHBTE: 51.4% vs. 46.0%, *p* = 0.452). While larger validation samples would enhance precision and narrow confidence intervals, the consistency of performance metrics (AUC 0.938, 95% CI: 0.884–0.992; calibration *χ*^2^ = 10.977, *p* = 0.203) supports preliminary generalizability. Future multicenter validation with larger cohorts is warranted to confirm these findings. Fifth, inter-rater and intra-rater reliability for MSKUS assessments were not formally evaluated in this study, though examinations were conducted by a single experienced physician following international guidelines (17). Future studies should incorporate formal reliability testing to further strengthen methodological rigor. Sixth, some clinically relevant variables (BMI, hyperglycemia, anxiety) were excluded from the final model despite known associations with neck–shoulder pain in previous literature. This reflects statistical selection based on predictive value for ROM limitation rather than pain presence—these variables showed no independent association with limited ROM in multivariate analysis (*p* > 0.05). Specifically, in our cohort, BMI (median 24.51 vs. 24.83 kg/m^2^, *p* = 0.152), hyperglycemia (11.0% vs. 13.6%, *p* = 0.562), and anxiety (13.0% vs. 9.1%, *p* = 0.365) did not differ significantly between limited ROM and normal ROM groups in univariate analysis, and were not retained in the final multivariate model. As detailed in Section 4.2, this divergence likely occurs because these factors influence nociceptive processing without mechanically restricting joint mobility, whereas our model prioritizes structural predictors (GLE, SASDBE, LHBTE) and mechanical exposures (DOAD) that physically constrain range of motion. This selection enhances model parsimony and clinical interpretability, though future studies might explore interaction effects between metabolic/psychological and structural factors in larger cohorts.

### 4.5 Future directions

Given the cross-sectional nature of this study, subsequent research should prioritize prospective longitudinal cohorts to establish temporal causality and determine the optimal timing for intervention. Future studies should also validate this model in diverse occupational settings (including male nurses and non-tertiary hospitals) and explore dynamic MSKUS assessment during arm elevation to capture functional impingement phenomena. Integration of biochemical markers (e.g., inflammatory cytokines) might enhance predictive precision. Randomized trials evaluating targeted physiotherapy or ergonomic modifications guided by this risk stratification tool would establish its clinical effectiveness.

## 5 Conclusion

This study presents a prediction model that combines clinical data with MSKUS assessment to identify nursing staff at high risk of limited shoulder ROM. The nomogram facilitates personalized risk stratification and may inform targeted preventive strategies to preserve shoulder function in this essential workforce.

## Data Availability

The original contributions presented in the study are included in the article/supplementary material, further inquiries can be directed to the corresponding author.
